# Chronic Health Conditions and Longitudinal Employment in Survivors of Childhood Cancer

**DOI:** 10.1001/jamanetworkopen.2024.10731

**Published:** 2024-05-10

**Authors:** Neel S. Bhatt, Pamela Goodman, Wendy M. Leisenring, Gregory T. Armstrong, Eric J. Chow, Melissa M. Hudson, Kevin R. Krull, Paul C. Nathan, Kevin C. Oeffinger, Leslie L. Robison, Anne C. Kirchhoff, Daniel A. Mulrooney

**Affiliations:** 1 Fred Hutchinson Cancer Center, Seattle, Washington; 2 University of Washington School of Medicine, Seattle; 3 St Jude Children’s Research Hospital, Memphis, Tennessee; 4 The Hospital for Sick Children, The University of Toronto, Toronto, Ontario, Canada; 5 Duke Cancer Institute, Durham, North Carolina; 6 Huntsman Cancer Institute, and Department of Pediatrics, University of Utah, Salt Lake City

## Abstract

**Importance:**

Employment is an important factor in quality of life and provides social and economic support. Longitudinal data on employment and associations with chronic health conditions for adult survivors of childhood cancer are lacking.

**Objective:**

To evaluate longitudinal trends in employment among survivors of childhood cancer.

**Design, Setting, and Participants:**

Retrospective cohort study of 5-year cancer survivors diagnosed at age 20 years or younger between 1970 and 1986 enrolled in the multi-institutional Childhood Cancer Survivor Study (CCSS). Sex-stratified employment status at baseline (2002 to 2004) and follow-up (2014 to 2016) was compared with general population rates from the Behavioral Risk Factor Surveillance System cohort. Data were analyzed from July 2021 to June 2022.

**Exposures:**

Cancer therapy and preexisting and newly developed chronic health conditions.

**Main Outcomes and Measures:**

Standardized prevalence ratios of employment (full-time or part-time, health-related unemployment, unemployed, not in labor force) among adult (aged ≥25 years) survivors between baseline and follow-up compared with the general population. Longitudinal assessment of negative employment transitions (full-time to part-time or unemployed at follow-up).

**Results:**

Female participants (3076 participants at baseline; 2852 at follow-up) were a median (range) age of 33 (25-53) years at baseline and 42 (27-65) years at follow-up; male participants (3196 participants at baseline; 2557 at follow-up) were 33 (25-54) and 43 (28-64) years, respectively. The prevalence of full-time or part-time employment at baseline and follow-up was 2215 of 3076 (71.3%) and 1933 of 2852 (64.8%) for female participants and 2753 of 3196 (85.3%) and 2079 of 2557 (77.3%) for male participants, respectively, with declining standardized prevalence ratios over time (female participant baseline, 1.01; 95% CI, 0.98-1.03; follow-up, 0.94; 95% CI, 0.90-0.98; *P* < .001; male participant baseline, 0.96; 95% CI, 0.94-0.97; follow-up, 0.92; 95% CI, 0.89-0.95; *P* = .02). While the prevalence of health-related unemployment increased (female participants, 11.6% to 17.2%; male participants, 8.1% to 17.1%), the standardized prevalence ratio remained higher than the general population and declined over time (female participant baseline, 3.78; 95% CI, 3.37-4.23; follow-up, 2.23; 95% CI, 1.97-2.51; *P* < .001; male participant baseline, 3.12; 95% CI, 2.71-3.60; follow-up, 2.61; 95% CI, 2.24-3.03; *P* = .002). Among survivors employed full-time at baseline (1488 female participants; 1933 male participants), 285 female participants (19.2%) and 248 male participants (12.8%) experienced a negative employment transition (median [range] follow-up, 11.5 [9.4-13.8] years). Higher numbers and grades of chronic health conditions were significantly associated with these transitions.

**Conclusions and Relevance:**

In this retrospective analysis of adult survivors of childhood cancer, significant declines in employment and increases in health-related unemployment among cancer survivors compared with the general population were identified. A substantial portion of survivors in the midcareer age range fell out of the workforce. Awareness among clinicians, caregivers, and employers may facilitate clinical counseling and occupational provisions for supportive work accommodations.

## Key Points

**Question:**

Can long-term childhood cancer survivors sustain employment as they get older, and how are their employment changes associated with chronic health conditions?

**Findings:**

In this cohort study, among adults previously treated for a childhood cancer who attained full-time employment 25 years following successful treatment, nearly 30% to 40% either died, became unemployed, or moved to part-time employment or left the labor force within the subsequent decade. Chronic health conditions with varying onset and severity were significantly associated with employment transitions.

**Meaning:**

These findings suggest efforts to prevent or mitigate chronic health conditions and improve workplace provisions for cancer survivors are needed.

## Teaser

This cohort study evaluates longitudinal trends in employment among survivors of childhood cancer.

## Introduction

With advances in therapies and supportive care, 5-year survival rates for pediatric cancer now exceed 85%,^1^ with over half a million adult survivors of childhood cancer living in the US.^2^ As these numbers continue to increase, so has the understanding of the long-term health complications survivors face after completion of therapy.^3,4,5,6,7,8,9^ Additionally, psychosocial sequelae following cancer can further impact physical and mental health, social interactions, education, sustained employment, and insurance access.

Beyond the economic implications, employment after completion of therapy is an important factor in social integration and quality of life for survivors coping with treatment-related toxic effects. According to the Surveillance Epidemiology and End Results Program data, two-thirds of childhood cancer survivors living in the US by 2011 were within the eligible ages of work force participation. Employment disruptions are associated with a higher burden of material and psychological financial hardship, and studies have reported higher health-related unemployment rates among childhood cancer survivors compared with controls.^10,11,12,13^ However, most studies are limited due to their cross-sectional design and few have assessed associations between employment and chronic health conditions. A prior study from the Childhood Cancer Survivor Study (CCSS) reported that at a mean (range) age of 27 (18-48) years, 62% of survivors had at least 1 chronic health condition and 27% had a severe or life-threatening condition,^8^ which represents a significant health burden during the key years of workforce participation. While chronic health conditions and the resultant functional impairments may impact survivors’ abilities to keep up with workplace demands, no studies have assessed associations between chronic health conditions and changes in employment status over time. To address this knowledge gap, we aimed to describe the employment status of adult survivors of childhood cancer participating in the CCSS, specifically longitudinal changes, including health-related reasons for unemployment and associations with the development of chronic health conditions during survivorship.

## Methods

### Study Design and Participants

The CCSS is a National Cancer Institute–funded multi-institutional retrospective cohort study with longitudinal follow-up of 5-year survivors of childhood cancer diagnosed when aged 20 years or younger between January 1, 1970, and December 31, 1999, at 1 of 31 institutions in North America. Study details have been previously described.^14,15^ Initially established in 1994 and composed of childhood cancer survivors diagnosed between 1970 and 1986 (original cohort), the CCSS was subsequently expanded to include survivors diagnosed between 1987 and 1999 (expansion cohort). Treatment details were abstracted from institutional medical records and survivors have provided self-reported data on sociodemographics, health behaviors, chronic health conditions, and quality of life through periodic surveys. Our analysis focused on the survivors from the original cohort due to the length of available follow-up and our aim of understanding changes in employment status over time. To achieve this, we first conducted a cross-sectional analysis of adult participants (aged ≥25 years), who answered either the 2002 to 2004 (baseline) or 2014 to 2016 (follow-up) survey and had an available US address. Next, we restricted the analysis to the subset of survivors who responded to both the baseline and follow-up surveys to study the longitudinal changes in employment and associations with chronic health conditions. This analysis was not restricted to those with an available US address. The CCSS was approved by the institutional review boards at all participating sites, and participants provided written informed consent. This study followed Strengthening the Reporting of Observational Studies in Epidemiology (STROBE) reporting guidelines for observational studies.

### Outcomes

Self-reported employment categories captured from the CCSS surveys included full-time (defined as working ≥30 hours per week), part-time (defined as working <30 hours per week), unable to work due to illness or disability (health-related unemployment), unemployed and looking for work, or not part of the labor force (ie, retired, student, or caring for home or family). For each category, general population rates matched by sex, race and ethnicity, census bureau division,^16^ age, and survey year were obtained from the Behavior Risk Factor Surveillance System (BRFSS) (eTable 1 in Supplement 1). BRFSS is a nationwide longitudinal health survey of the US adult population sponsored by the Centers for Disease Control and Prevention and other federal agencies conducted in all 50 states, the District of Columbia, and 3 territories, containing data on more than 400 000 adults interviewed each year.^17^ BRFSS uses proportional fitting weighting using demographic and geographic factors to increase the representativeness of the estimates. BRFSS data were used for the employment status category comparison due to their similarity with the CCSS surveys and availability of data for the time period being studied. For the cross-sectional analysis, we combined full-time and part-time employment due to the limited number of participants reporting part-time employment. For the longitudinal analysis, the outcome was a negative employment transition, defined as a change in employment from full-time at baseline to either part-time employment, unemployed and looking for work, or health-related unemployment.

### Factors Associated With Risk

Chronic health conditions were self-reported conditions with first age of onset ascertained from CCSS surveys and were assigned grades per the National Cancer Institute’s Common Terminology Criteria for Adverse Events version 4.03 as mild (grade 1), moderate (grade 2), severe or disabling (grade 3), life-threatening (grade 4), or fatal (grade 5). The method of grading chronic health conditions within the CCSS cohort has been previously described.^6,8^ Subsequent neoplasms (SMN) were confirmed by pathology report review.

### Statistical Analysis

Sociodemographic and clinical characteristics were described using means (SDs) and medians (ranges) for continuous variables and frequencies (percentages) for categorical variables. Due to anticipated sex differences in employment status, analyses were stratified by sex and adjusted for age at diagnosis, age at survey, and self-reported race and ethnicity. Race and ethnicity were assessed in this study because of potential differences in unemployment by race and ethnicity. Participants were grouped into non-Hispanic White and other (includes Black, American Indian or Native Alaskan, Asian or Pacific Islander, and mixed races) because of the limited number of participants in categories other than non-Hispanic White. For the cross-sectional analyses, prevalence and standardized prevalence ratio with 95% CI for employment status categories were estimated. Standardized prevalence ratio was defined as the prevalence of each employment status category for survivors divided by the expected general population prevalence of that category from the BRFSS, matched by sex, race and ethnicity, census bureau division, age at survey, and calendar year of survey. Canadian survivors (10 participants), who were not included in BRFSS, were excluded in these comparisons. For each employment category, standardized prevalence ratios were compared between the baseline and follow-up time points. For the longitudinal analyses, among survivors who reported full-time employment at baseline, we evaluated the associations of the timing (before baseline and between baseline and follow-up), number (≥2 vs 0-1), severity (grade 2 and grades 3 to 4 vs grades 0 to 1), and separately, the type of chronic health conditions, with negative employment transitions at the follow-up survey using generalized log-linear regression models to calculate adjusted prevalence ratios and 95% CIs. Additionally, we described the characteristics and subsequent status of survivors who reported health-related unemployment at baseline. Given the small number of survivors whose employment status changed in this group, multivariable analyses of persistent health-related unemployment were not conducted. To account for missing data due to nonparticipation, all analyses used inverse probability weighting (IPW) at each relevant survey among otherwise eligible individuals (alive at the time the survey was sent). Among those eligible, weights were calculated from participation probabilities at each survey estimated from logistic regression models with covariates, including cancer diagnosis, age at diagnosis, year of diagnosis, sex, race, ethnicity, treating institution, and age survey was sent; as of the most recently answered survey, covariates were household income, health insurance, smoking, body mass index, attained education, physical activity level, and prior chronic health conditions. Analyses used robust sandwich estimators of variance. Additional analyses that did not use IPW did not differ qualitatively from those using weights and are not shown. Otherwise, if a participant was missing a variable needed for a specific analysis, they were excluded from that analysis. *P* values of .05 or less were considered statistically significant and adjustments were not made for multiple comparisons. All analyses were completed using SAS version 9.4 (SAS Institute) and graphs created in R version 3.5.3 (R Project for Statistical Computing). The analysis was conducted from July 2021 to June 2022.

## Results

Among CCSS survivors over the age of 25 years, a total of 6272 survivors responded to the baseline survey (3196 male participants, 5665 White non-Hispanic participants, and 607 participants of other races and ethnicities) and 5409 survivors responded to the follow-up survey (2852 female participants, 4908 White non-Hispanic participants, and 501 participants of other races and ethnicities) (Table 1). A total of 4291 survivors (2243 female participants and 2048 male participants) responded to both. Out of those who responded to the baseline survey, 1981 (31.6%) did not respond to the follow-up survey due to either active or passive refusal, lost to follow-up status, or death (eFigure in Supplement 1; Table 1). Characteristics of the survivors who responded to either the baseline or follow-up were very similar to those who answered both surveys (eTable 2 in Supplement 1). Among female participants, the median (range) age at baseline survey was 33 (25-53) years and 42 (27-65) years at follow-up. Male participants had a median (range) age of 33 (25-54) years and 43 (28-64) years at baseline and follow-up, respectively. Acute lymphoblastic leukemia, sarcomas (bone and soft tissue), and Hodgkin lymphoma were the most common diagnoses at both time points.

**Table 1.** Sex-Stratified Demographic and Treatment Characteristics of Childhood Cancer Survivors

Characteristic	Participants, No. (%)		
	Answered baseline and/or follow-up surveys^a^		Working full-time at baseline and responded to follow-up
	Baseline	Follow-up	
Female sex			
Participants, total No.	3076^b^	2852	1337
Age at diagnosis, median (range), y	9 (0-20)	7 (0-20)	9 (0-20)
Age at survey, median (range), y	33 (25-53)	42 (27-65)	45 (34-65)
Years since diagnosis, median (range)	24.9 (16.2-35.2)	34.6 (27.6-46.4)	36.3 (27.6-46.4)
Age at diagnosis, y			
0-4	852 (27.7)	1196 (41.9)	367 (27.4)
5-9	718 (23.3)	566 (19.9)	302 (22.6)
10-14	791 (25.7)	585 (20.5)	361 (27.0)
15-20	715 (23.2)	505 (17.7)	307 (23.0)
Race and ethnicity			
White non-Hispanic	2762 (89.8)	2568 (90.0)	1172 (87.7)
Other^c^	314 (10.2)	284 (10.0)	115 (8.6)
Unknown	0	0	50 (3.7)
Health insurance status			
Yes	2769 (90.5)	2684 (94.5)	1225 (92.1)
No	287 (9.4)	154 (5.4)	50 (3.8)
Canadian resident	4 (0.1)	1 (<0.1)	55 (4.1)
Not reported	16	13	7
Primary cancer diagnosis			
Acute lymphoblastic leukemia	886 (28.8)	903 (31.7)	410 (30.7)
Acute myeloid leukemia	88 (2.9)	90 (3.2)	35 (2.6)
Astrocytoma	238 (7.7)	200 (7.0)	70 (5.2)
Medulloblastoma	63 (2.1)	56 (2.0)	22 (1.7)
Hodgkin lymphoma	530 (17.2)	356 (12.5)	229 (17.1)
Non-Hodgkin lymphoma	171 (5.6)	135 (4.7)	73 (5.5)
Kidney (Wilms)	257 (8.4)	323 (11.3)	132 (9.9)
Neuroblastoma	151 (4.9)	220 (7.7)	62 (4.6)
Sarcomas	617 (20.1)	495 (17.4)	277 (20.7)
Soft tissue sarcoma	298 (9.7)	254 (8.9)	143 (10.7)
Ewing sarcoma	98 (3.2)	71 (2.5)	38 (2.8)
Osteosarcoma	221 (7.2)	170 (6.0)	96 (7.2)
Other diagnoses	75 (2.4)	74 (2.6)	27 (2.0)
Other leukemia	21 (0.7)	20 (0.7)	7 (0.5)
Other CNS tumors	41 (1.3)	40 (1.7)	10 (0.7)
Other bone tumors	13 (0.4)	14 (0.5)	10 (0.7)
Treatment combinations			
No surgery, chemotherapy, or radiation	6 (0.2)	5 (0.2)	2 (0.2)
Surgery only	198 (7.1)	222 (8.4)	87 (7.0)
Chemotherapy only	204 (7.3)	284 (10.8)	93 (7.5)
Radiation only	6 (0.2)	6 (0.2)	3 (0.2)
Surgery plus chemotherapy	408 (14.7)	484 (18.4)	203 (16.3)
Surgery plus radiation	419 (15.1)	317 (12.1)	167 (13.4)
Chemotherapy plus radiation	523 (18.8)	463 (17.6)	241 (19.2)
Surgery plus chemotherapy plus radiation	1015 (36.5)	850 (32.3)	450 (36.1)
Age at survey, y			
25-34	1719 (55.9)	490 (17.2)	1 (0.1)
35-44	1141 (37.1)	1224 (42.9)	643 (48.1)
≥45	216 (7.0)	1138 (39.9)	693 (51.8)
Male sex			
Participants, total No.	3196^d^	2557	1712
Age at diagnosis, median (range), y	9 (0-20)	7 (0-20)	10 (0-20)
Age at survey, median (range), y	33 (25-54)	43 (28-64)	45 (35-64)
Years since diagnosis, median (range)	24.6 (16.1-34.6)	34.5 (27.6-46.7)	36.0 (27.7-46.7)
Age at diagnosis, y			
0-4	829 (25.9)	985 (38.5)	404 (23.6)
5-9	840 (26.3)	577 (22.6)	441 (25.8)
10-14	811 (25.4)	543 (21.2)	475 (27.7)
15-20	716 (22.4)	452 (17.7)	392 (22.9)
Race and ethnicity			
White non-Hispanic	2903 (90.8)	2340 (91.5)	1521 (88.8)
Other^c^	293 (9.2)	217 (8.5)	114 (11.2)
Unknown	0	0	77 (4.5)
Health insurance status			
Yes	2763 (87.1)	2355 (92.5)	1529 (89.8)
No	405 (12.8)	191 (7.5)	107 (6.3)
Canadian resident	6 (0.2)	1 (<0.1)	67 (3.9)
Not reported	22	10	9
Primary cancer diagnosis			
Acute lymphoblastic leukemia	873 (27.3)	783 (30.6)	486 (28.4)
Acute myeloid leukemia	66 (2.1)	55 (2.2)	30 (1.8)
Astrocytoma	215 (6.7)	151 (5.9)	89 (5.2)
Medulloblastoma	74 (2.3)	62 (2.4)	31 (1.8)
Hodgkin lymphoma	527 (16.5)	316 (12.4)	281 (16.4)
Non-Hodgkin lymphoma	385 (12.1)	278 (10.9)	217 (12.7)
Kidney (Wilms)	195 (6.1)	222 (8.7)	99 (5.8)
Neuroblastoma	113 (3.5)	155 (6.1)	59 (3.4)
Sarcomas	637 (19.9)	440 (17.2)	359 (21.0)
Soft tissue sarcoma	327 (10.2)	228 (8.9)	181 (10.6)
Ewing sarcoma	108 (3.4)	70 (2.7)	60 (3.5)
Osteosarcoma	202 (6.3)	142 (5.5)	118 (6.9)
Other diagnoses	111 (3.5)	95 (3.7)	61 (3.6)
Other leukemia	31 (0.9)	28 (1.1)	22 (1.3)
Other CNS tumors	69 (2.1)	60 (2.3)	34 (2.0)
Other bone tumors	11 (0.3)	7 (0.3)	5 (0.3)
Treatment combinations			
No surgery, chemotherapy, or radiation	6 (0.2)	8 (0.4)	4 (0.3)
Surgery only	187 (6.7)	175 (7.6)	108 (7.0)
Chemotherapy only	102 (3.7)	135 (5.9)	59 (3.8)
Radiation only	12 (0.4)	9 (0.4)	9 (0.6)
Surgery plus chemotherapy	525 (18.8)	498 (21.7)	299 (19.4)
Surgery plus radiation	362 (13.0)	241 (10.5)	193 (12.5)
Chemotherapy plus radiation	250 (9.0)	215 (9.4)	155 (10.0)
Surgery plus chemotherapy plus radiation	1346 (48.2)	1012 (44.1)	718 (46.5)
Age at survey, y			
25-34	1800 (56.3)	364 (14.2)	0 (0)
35-44	1184 (37.1)	1125 (44.0)	772 (45.1)
≥45	212 (6.6)	1068 (41.8)	940 (54.9)

Abbreviation: CNS, central nervous system.

^a^ Participants who responded to either baseline or follow-up surveys were restricted to those with available US address because of the comparison with Behavior Risk Factor Surveillance System cohort; participants who responded to both baseline and follow-up surveys were not restricted to those with available US address.

^b^ Of the 3076 female survivors who answered baseline, 2243 also responded to the follow-up survey.

^c^ The other group includes American Indian or Alaska Native, Asian or Pacific Islander, Black, and mixed races.

^d^ Of the 3196 male survivors who answered baseline, 2048 also responded to the follow-up survey.

### Cross-Sectional Analysis

**Figure 1.**
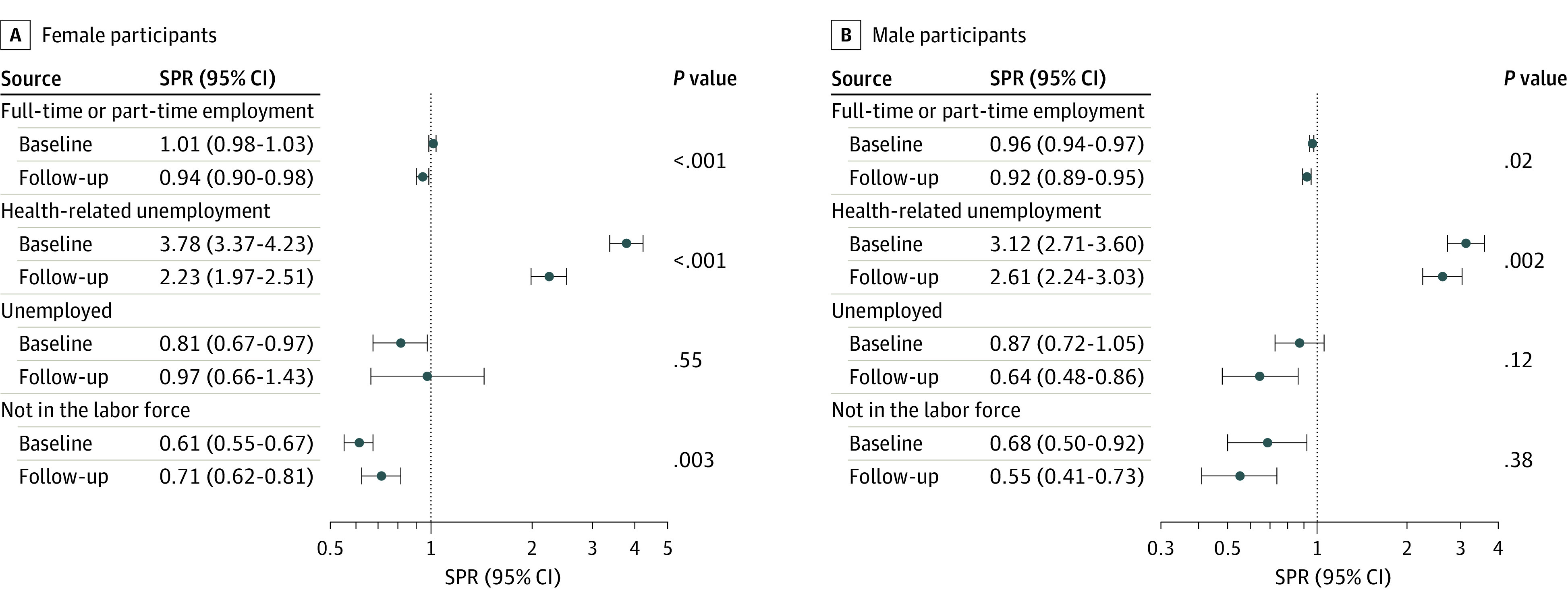
Standardized Prevalence Ratios (SPRs) and 95% CIs for Employment Status Categories, Among Childhood Cancer Survivors Compared With the General Population Employment status categories include full-time employment defined as working 30 or more hours per week; part-time employment defined as working less than 30 hours per week; health-related unemployment defined as inability to work due to illness or disability; unemployed and looking for work; and not part of the labor force including survivors who are retired, students, or caring for home or family.

The prevalence of full-time or part-time employment for female participants at baseline and follow-up was 2215 of 3076 (71.3%) and 1933 of 2852 (64.8%), respectively, and for male participants, 2753 of 3196 (85.3%) and 2079 of 2557 (77.3%). Between baseline and follow-up, the standardized prevalence ratio of full-time or part-time employment declined for both sexes (female participant baseline, 1.01; 95% CI, 0.98-1.03; follow-up, 0.94; 95% CI, 0.90-0.98; male participant baseline, 0.96; 95% CI, 0.94-0.97; follow-up, 0.92; 95% CI, 0.89-0.95) (Figure 1; eTable 3 in Supplement 1). The prevalence of survivors reporting health-related unemployment increased from baseline to follow-up (328 of 3076 [11.6%] to 422 of 2852 [17.2%] among female participants and 244 of 3196 [8.1%] to 320 of 2557 [17.1%] among male participants). The standardized prevalence ratio for health-related unemployment, which remained significantly higher compared with the general population, was noted to decline for female participants (baseline, 3.78; 95% CI, 3.37-4.23; follow-up, 2.23; 95% CI, 1.97-2.51) and male participants (baseline, 3.12; 95% CI, 2.71-3.60; follow-up, 2.61; 95% CI, 2.24-3.03) over time. No significant difference in standardized prevalence ratio of unemployed and looking for work was noted between baseline and follow-up for either sex. The standardized prevalence ratio for female participants who were not a part of the labor force increased (baseline, 0.61; 95% CI, 0.55-0.67; follow-up, 0.71; 95% CI, 0.62-0.81; *P* = .003), but not for male participants (baseline, 0.68; 95% CI, 0.50-0.92; follow-up, 0.55; 95% CI, 0.41-0.73; *P* = .38).

### Longitudinal Analysis

**Figure 2.**
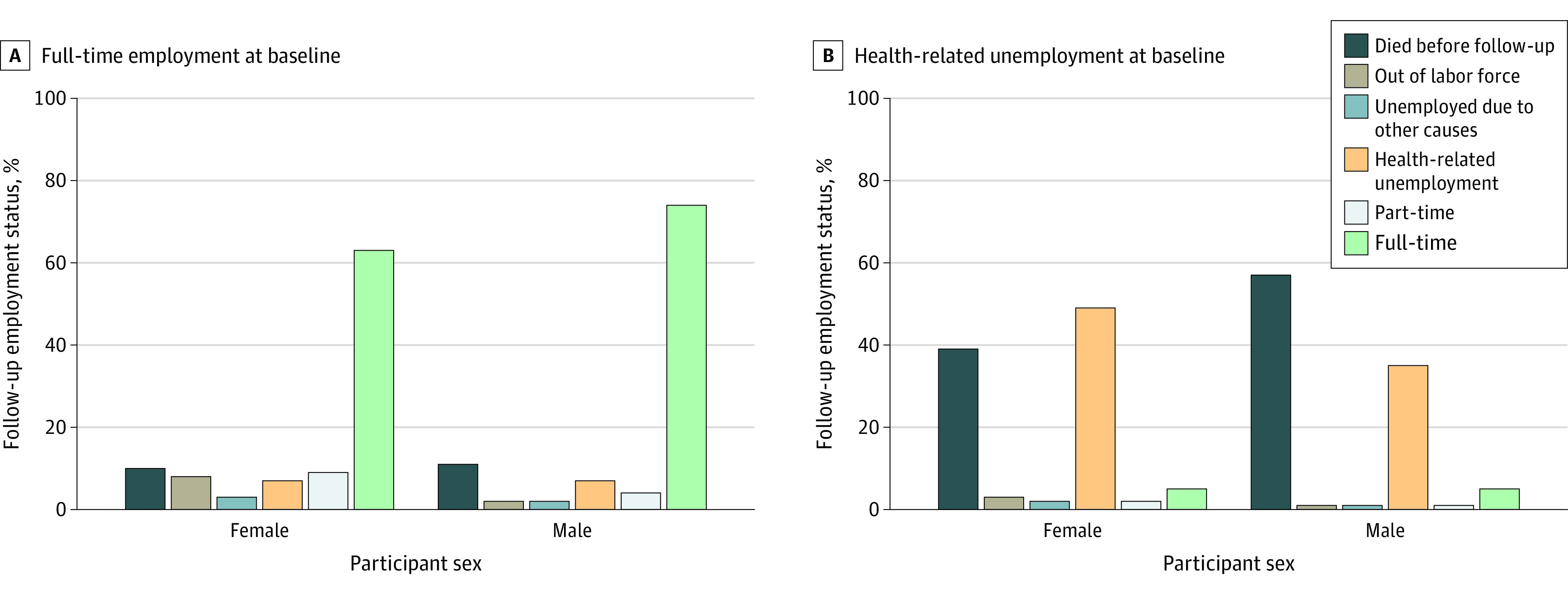
Status of Survivors at Follow-Up Among Those Reporting Full-Time Employment or Health-Related Unemployment at Baseline Denominators for each bar include the number of female or male survivors who responded as indicated (full-time employment or health-related unemployment) on the baseline survey and either answered the follow-up or died before the follow-up.

Among survivors working full-time at baseline with a known vital status and, if alive, known employment status at follow-up (1488 female participants and 1933 male participants), 925 female participants (62.2%) and 1408 male participants (72.8%) remained employed full-time, 134 (9.0%) and 71 (3.7%) transitioned to part-time employment, 151 (10.1%) and 171 (8.8%) became unemployed for any reason, and 127 (8.5%) and 56 (2.8%) left the labor force, respectively. Median (range) follow-up interval was 11.5 (9.4-13.8) years. For both male and female participants, 6.8% (101 female and 132 male participants) became unemployed for health-related reasons. Deaths before follow-up occurred in 151 female participants (10.2%) and 221 male participants (11.4%) (Figure 2). Overall, among survivors employed full-time at baseline, who remained alive and reported employment status, 285 female participants (21.3%) and 248 male participants (14.5%) had a negative employment transition by the follow-up survey.

Among 465 living survivors reporting health-related unemployment at baseline and with a known vital status and, if alive, known employment status at follow-up (250 female participants and 215 male participants), 124 female participants (49.6%) and 75 male participants (34.9%) still reported health-related unemployment at follow-up (Figure 2). A total of 97 female participants (38.8%) and 122 male participants (56.7%) died before follow-up. Among those who died, grade 3 to 4 SMNs (23 female participants [23.7%] and 20 male participants [16.4%]) and cardiac conditions (15 female participants [15.5%] and 18 male participants [14.8%]) were the most common chronic health conditions (eTable 4 in Supplement 1).

**Figure 3.**
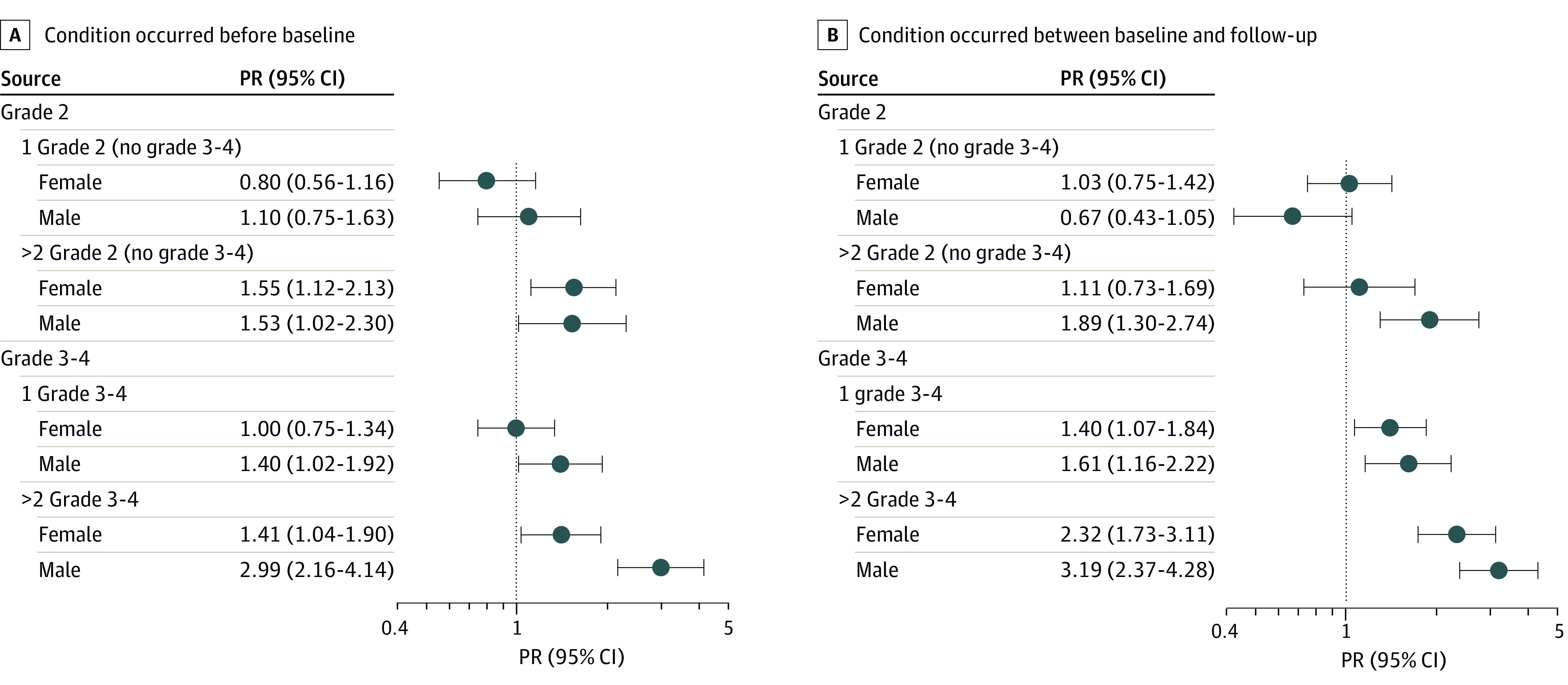
Prevalence Ratios (PRs) for a Negative Employment Transition Based on Presence of Chronic Health Conditions: Associations With Timing, Severity, and Number of Chronic Health Conditions

Among participants working full-time at baseline who remained alive and responded to follow-up (1337 female participants and 1712 male participants) (Table 1; eTable 5 in Supplement 1), after adjusting for race, ages at diagnosis and follow-up, both higher grade and number of chronic health conditions were associated with negative employment transitions (Figure 3; eTable 6 in Supplement 1). For female survivors, the presence of 2 or more grade 2 chronic health conditions (prevalence ratio, 1.55; 95% CI, 1.12-2.13; *P* = .01) or 2 or more grade 3 to 4 chronic health conditions (prevalence ratio, 1.41; 95% CI, 1.04-1.90; *P* = .03) before baseline and 1 (prevalence ratio, 1.40; 95% CI, 1.07-1.84; *P* = .02) or 2 or more grade 3 to 4 chronic health conditions (prevalence ratio, 2.32; 95% CI, 1.73-3.11; *P* < .001) between baseline and follow-up were associated with negative employment transitions. In contrast, among male participants, 2 or more grade 2 (prevalence ratio, 1.53; 95% CI, 1.02-2.30; *P* = .04), 1 grade 3 to 4 (prevalence ratio, 1.40; 95% CI, 1.02-1.92; *P* = .04), and 2 or more grade 3 to 4 conditions (prevalence ratio, 2.99; 95% CI, 2.16-4.14; *P* < .001) before baseline were associated with negative transitions in employment. Development of 1 grade 3 to 4 and 2 or more grade 2 or 3 to 4 conditions between baseline and follow-up were also associated with a negative employment transition for male participants.

Survivors with grade 2 or 3 to 4 neurologic conditions acquired before baseline or between baseline and follow-up had a higher risk of moving from full-time to part-time or being unemployed compared with those without these conditions (Table 2; eTable 5 in Supplement 1). Male and female participants with grade 3 to 4 respiratory conditions before baseline and cardiac and musculoskeletal conditions acquired between baseline and follow-up were also at higher risk of moving to part-time or unemployed or unable to work. Additional variables for male participants (but not female participants) included grade 2 vision and endocrine conditions.

**Table 2.** Association Between Type and Timing of Chronic Health Conditions and Risk of Negative Employment Transitions^a^

Factor associated with risk	Female participants		Male participants	
	Prevalence ratio (95% CI)	*P* value	Prevalence ratio (95% CI)	*P* value
Age at diagnosis				
0-4	0.95 (0.65-1.38)	.77	1.27 (0.83-1.93)	.27
5-9	0.94 (0.66-1.35)	.75	1.43 (1.00-2.03)	.049
10-14	0.95 (0.70-1.29)	.75	1.32 (0.96-1.82)	.09
≥15	1 [Reference]	NA	1 [Reference]	NA
Race and ethnicity				
Other^b^	1.23 (0.92-1.65)	.16	1.00 (0.70-1.42)	.99
Non-Hispanic White	1 [Reference]	NA	1 [Reference]	NA
Age at survey, y				
<45	1.15 (0.88-1.49)	.31	0.73 (0.66-0.96)	.02
≥45	1 [Reference]	NA	1 [Reference]	NA
Visual				
Before baseline				
No grade 2-4	NA	NA	1 [Reference]	NA
Maximum grade 2	NA	NA	2.30 (1.56-3.39)	<.001
Maximum grade 3-4	NA	NA	1.46 (0.97-2.19)	.07
Between baseline and follow-up				
No grade 2-4	NA	NA	1 [Reference]	NA
Maximum grade 2	NA	NA	1.67 (1.07-2.61)	.02
Maximum grade 3-4	NA	NA	0.67 (0.31-1.49)	.33
Endocrine				
Before baseline				
No grade 2-4	NA	NA	1 [Reference]	NA
Maximum grade 2	NA	NA	1.42 (1.07-1.87)	.01
Maximum grade 3-4	NA	NA	1.30 (0.94-1.81)	.12
Between baseline and follow-up				
No grade 2-4	NA	NA	1 [Reference]	NA
Maximum grade 2	NA	NA	1.73 (1.32-2.26)	<.001
Maximum grade 3-4	NA	NA	1.32 (0.92-1.91)	.13
Respiratory				
Before baseline				
No grade 2-4	1 [Reference]	NA	1 [Reference]	NA
Maximum grade 2	1.40 (1.01-1.94)	.04	0.94 (0.55-1.61)	.82
Maximum grade 3-4	2.46 (1.48-4.10)	.001	2.06 (1.20-3.54)	.01
Between baseline and follow-up				
No grade 2-4	1 [Reference]	NA	1 [Reference]	NA
Maximum grade 2	1.16 (0.71-1.90)	.55	0.60 (0.13-2.74)	.51
Maximum grade 3-4	1.26 (0.61-2.62)	.54	2.82 (1.43-5.55)	.003
Cardiac				
Before baseline				
No grade 2-4	1 [Reference]	NA	1 [Reference]	NA
Maximum grade 2	0.97 (0.74-1.28)	.83	0.89 (0.67-1.18)	.40
Maximum grade 3-4	0.77 (0.54-1.10)	.15	1.22 (0.87-1.70)	.24
Between baseline and follow-up				
No grade 2-4	1 [Reference]	NA	1 [Reference]	NA
Maximum grade 2	0.93 (0.72-1.21)	.60	0.96 (0.72-1.28)	.78
Maximum grade 3-4	1.77 (1.35-2.32)	<.001	1.86 (1.44-2.39)	<.001
Musculoskeletal				
Before baseline				
No grade 2-4	1 [Reference]	NA	1 [Reference]	NA
Maximum grade 2	2.53 (0.44-14.45)	.30	2.33 (0.90-6.05)	.08
Maximum grade 3-4	1.00 (0.67-1.49)	.99	1.00 (0.59-1.71)	.99
Between baseline and follow-up				
No grade 2-4	1 [Reference]	NA	1 [Reference]	NA
Maximum grade 3-4	1.66 (0.99-2.80)	.06	2.72 (1.82-4.07)	<.001
Neurologic				
Before baseline				
No grade 2-4	1 [Reference]	NA	1 [Reference]	NA
Maximum grade 2	1.67 (1.24-2.24)	.001	1.76 (1.27-2.43)	.001
Maximum grade 3-4	1.83 (1.28-2.62)	.001	1.67 (1.17-2.38)	.01
Between baseline and follow-up				
No grade 2-4	1 [Reference]	NA	1 [Reference]	NA
Maximum grade 2	2.50 (1.78-3.52)	<.001	2.29 (1.68-3.13)	<.001
Maximum grade 3-4	2.93 (2.09-4.11)	<.001	3.91 (2.88-5.29)	<.001

Abbreviation: NA, not applicable.

^a^ All variables were examined; those with NA were nonsignificant chronic conditions that were removed during modelling.

^b^ The other group includes American Indian or Alaska Native, Asian or Pacific Islander, Black, and mixed races.

## Discussion

In what we believe to be the most comprehensive assessment to date of longitudinal employment among long-term survivors of childhood cancer, we identified significant declines in employment and increases in health-related unemployment among survivors compared with the general population. Importantly, a substantial portion, in the midcareer age range, fell out of the workforce. These negative employment transitions were associated with chronic health conditions, particularly with new onset and accumulation of multiple conditions over time. The increased prevalence and risks for chronic health conditions experienced by survivors of childhood cancer in early adulthood may impact long-term employment, with subsequent social and financial implications.

Employment is an important indicator of socioeconomic independence.^18^ Intermittent employment or underemployment can be a source of life-long economic instability. This is particularly concerning for survivors in the US, whose health insurance coverage is often tied to employment status despite enactment of the Patient Protection and Affordable Care Act (ACA).^19^ In our analysis, we noted that the standardized prevalence ratio of health-related unemployment declined for both female and male survivors from baseline to follow-up. While the reasons for the declining health-related unemployment standardized prevalence ratio are unclear, it is possible that the need for insurance coverage for health care access may have played a role, as the majority of insurance for working age adults in the US remains employer-based. Nonetheless, the standardized prevalence ratio for health-related unemployment remained higher than the general population at both time points. Employment difficulties among survivors have been shown to be associated with medical and nonmedical financial burdens, quality of life impairment, and limitations in adherence to medical management.^20,21,22,23^ Our findings highlight the lifelong effects cancer treatment can have on the socioeconomic dynamics important to maintaining long-term health and could be generalized to all living cancer survivors treated in the US. However, it is important to note that our analysis focusing on employment transitions from baseline to follow-up survey was restricted to those survivors who remained alive and responded to both of the surveys. In addition, many survivors may have died before the baseline survey, and we did not have any employment data for these survivors. Thus, it is important to consider the results of our study within the context of living survivors at the time points postdiagnosis at which we observed their employment.

Employment transitions among aging adult survivors and their relationship with chronic health conditions have not been previously described in a large, geographically diverse population such as the CCSS. In a recent analysis of employment status of long-term Nordic childhood cancer survivors (10 461 participants),^13^ the authors noted that 9.2% of survivors were unemployed due to health-related reasons by age 30 years, which increased to 11.6% by age 40 years and 14.3% by age 50 years. In our analysis, we specifically focused on employment transitions of survivors who were employed full-time or reporting health-related unemployment at baseline survey. We noted that a substantial proportion of those who were employed full-time at baseline transitioned to part-time employment or became unemployed, and most of those reporting health-related unemployment remained in that status a decade later or had died. These transitions could have important implications for long-term income potential, retirement planning, and ability to obtain and maintain health, life, or long-term care insurance. While the reasons for these transitions were unclear, given the associations with chronic health conditions, they could be due to functional impairments^24,25^ and resultant inability to keep up with work demands.^21,26,27^ Moreover, few cross-sectional studies have identified associations with vision, hearing, and neurologic systems and unemployment.^11,28^ A report from the St Jude Lifetime cohort noted significant associations between material hardship and chronic health conditions affecting the cardiovascular, neurologic, gastrointestinal, and reproductive systems, as well as SMN and limb amputations.^29^ Our analysis identified similar organ system associations with employment transitions but also found an increased risk with higher numbers and grades of conditions, and in particular with the new onset of conditions from the time of the baseline evaluation. Childhood cancer survivors are known to experience multiple chronic health conditions at an early age,^8,9,30^ suggesting a need for increased awareness to facilitate access to clinical and occupational counseling to reduce the risk of falling out of the workforce.

The current long-term follow-up guidelines from the Children’s Oncology Group (COG)^31^ recommends annual assessment of vocational progress for all cancer survivors, and more detailed assessments of employment-related challenges for high-risk survivors, such as those with multiple or high-grade comorbidities. A recently published evidence-based clinical practice guideline from the International Late Effects of Childhood Cancer Guidelines Harmonization Group (IGHG) further addressed concerns regarding employment.^32^ Similar to the COG, the IGHG recommends regular assessment of employment status and vocational planning, with particular attention to survivors with certain risk factors such as female sex, lower educational attainment, central nervous system (CNS) tumors, exposure to CNS-directed therapy, impaired neurocognitive function, subsequent neoplasm or cancer recurrence, or physical or psychological dysfunction. However, while these consensus-based recommendations provide guidance, with the large number of survivors falling out of the workforce in just over 10 years, intervention strategies are needed to prevent and mitigate chronic health conditions and their impact on employment in this population.^33,34^

### Limitations

Our findings should be considered in the context of some limitations. Since employment status and chronic health conditions were self-reported, there is a possibility of misclassification or social desirability bias. However, due to CCSS’s long-term relationship with participants, we do not believe that self-report bias could influence report of employment. Additionally, unemployment rates could be affected by external factors such as job availability, economic instability, seasonal employment, short-term employment, or the gig economy, among others. We attempted to adjust for these factors by comparing the prevalence of employment categories with general population data from the same geographical areas and years, among other matching factors, which was one of the strengths of our analysis. However, imperfect matching of employment categories between CCSS and BRFSS (eTable 1 in Supplement 1) could have led to potential measurement error. Some participant attrition was noted over time, potentially introducing some selection bias. However, we adjusted for potential dropout bias by using inverse probability weights, further accounting for any potential differences in characteristics between participants and nonparticipants. The sample size was not sufficient to calculate estimates stratified by racial or ethnic groups, although race and ethnicity were adjusted or accounted for in most of the analyses. Future research may be needed to better understand the impacts of race and ethnicity on long-term employment outcomes of minority and underserved populations. The definition of negative employment transition considered changes from full-time to either part-time employment, unemployed and looking for work, or health-related unemployment as equal, which depending on individual circumstances may not be the case. Additionally, the statistical power for some analyses may have been limited due to smaller sample sizes and/or number of events in some strata. Nonetheless, using the largest cohort of childhood cancer survivors in North America allowed identification of significant factors associated with long-term employment outcomes.

## Conclusions

While this study points to associations between chronic health conditions and employment over time among survivors, further research is needed to identify the implications of health-related unemployment on insurance access and income (including disability coverage), the development of social or financial independence, and health care utilization. Similarly, little is known about the ACA’s impact on employment transitions and insurance access among survivors. Additionally, for employed survivors, occupation and changes over time, missed work time, work productivity, and workplace accommodations should also be studied. A multidisciplinary approach that includes survivors, clinicians, and employers is likely needed to address long-term employment needs of cancer survivors. We anticipate future studies to test the feasibility and efficacy of interventions to reduce employment challenges and ultimately improve the quality of life of this population.

## Notes

Supplement 1. **eTable 1.** Mapping of Employment Status Variables From Childhood Cancer Survivor Study (CCSS) and Behavioral Risk Factor Surveillance System (BRFSS) Cohorts **eTable 2.** Sex Stratified Demographic and Treatment Characteristics of Childhood Cancer Survivors Diagnosed Between 1970 and 1986 and Aged 25 or more Years at the Time of Survey According to Their Response to Baseline and/or Follow-Up Surveys **eTable 3.** Prevalence, Standardized Prevalence Ratio (SPR), and 95% CI of Employment Status Among Childhood Cancer Survivors Enrolled in CCSS Original Cohort Relative to BRFSS Rates According to Time of Follow-Up Survey **eTable 4.** Characteristics of Childhood Cancer Survivors Diagnosed Between 1970 and 1986 and Aged 25 or more years at the Time of Survey Who Reported Health-Related Unemployment at Baseline **eTable 5.** Sex-Stratified Details on Overall and Organ-Specific Maximum Severity, Number, and Timing of Chronic Health Conditions of Childhood Cancer Survivors Who Reported Working Full-Time at Baseline and Responded to the Follow-Up Survey **eTable 6.** Overall Impact of Number and Severity of all Chronic Health Conditions (CHC) on the Risk of Negative Employment Transitions **eFigure.** Flow Diagram for the Study Population Derivation

Supplement 2.
Data Sharing Statement
