# Supplementary material for: Chronic Health Conditions and Longitudinal Employment in Survivors of Childhood Cancer
Source: JAMA Netw Open. 2024 May 10;7(5):e2410731. doi: 10.1001/jamanetworkopen.2024.10731 (PMC11087836; doi:10.1001/jamanetworkopen.2024.10731)
Supplement: Supplement 1. — eTable 1. Mapping of Employment Status Variables From Childhood Cancer Survivor Study (CCSS) and Behavioral Risk Factor Surveillance System (BRFSS) Cohorts eTable 2. Sex Stratified Demographic and Treatment Characteristics of Childhood Cancer Survivors Diagnosed Between 1970 and 1986 and Aged 25 or more Years at the Time of Survey According to Their Response to Baseline and/or Follow-Up Surveys eTable 3. Prevalence, Standardized Prevalence Ratio (SPR), and 95% CI of Employment Status Among Childhood Cancer Survivors Enrolled in CCSS Original Cohort Relative to BRFSS Rates According to Time of Follow-Up Survey eTable 4. Characteristics of Childhood Cancer Survivors Diagnosed Between 1970 and 1986 and Aged 25 or more years at the Time of Survey Who Reported Health-Related Unemployment at Baseline eTable 5. Sex-Stratified Details on Overall and Organ-Specific Maximum Severity, Number, and Timing of Chronic Health Conditions of Childhood Cancer Survivors Who Reported Working Full-Time at Baseline and Responded to the Follow-Up Survey eTable 6. Overall Impact of Number and Severity of all Chronic Health Conditions (CHC) on the Risk of Negative Employment Transitions eFigure. Flow Diagram for the Study Population Derivation [file jamanetwopen-e2410731-s001.pdf]

Supplemental Online Content

Bhatt NS, Goodman P, Leisenring WM, et al. Chronic health conditions and longitudinal employment in survivors of childhood cancer. *JAMA Netw Open*. 2024;7(5):e2410731. doi:10.1001/jamanetworkopen.2024.10731

eTable 1. Mapping of Employment Status Variables From Childhood Cancer Survivor Study (CCSS) and Behavioral Risk Factor Surveillance System (BRFSS) Cohorts
eTable 2. Sex Stratified Demographic and Treatment Characteristics of Childhood Cancer Survivors Diagnosed Between 1970 and 1986 and Aged 25 or more Years at the Time of Survey According to Their Response to Baseline and/or Follow-Up Surveys
eTable 3. Prevalence, Standardized Prevalence Ratio (SPR), and 95% CI of Employment Status Among Childhood Cancer Survivors Enrolled in CCSS Original Cohort Relative to BRFSS Rates According to Time of Follow-Up Survey
eTable 4. Characteristics of Childhood Cancer Survivors Diagnosed Between 1970 and 1986 and Aged 25 or more years at the Time of Survey Who Reported Health-Related Unemployment at Baseline
eTable 5. Sex-Stratified Details on Overall and Organ-Specific Maximum Severity, Number, and Timing of Chronic Health Conditions of Childhood Cancer Survivors Who Reported Working Full-Time at Baseline and Responded to the Follow-Up Survey
eTable 6. Overall Impact of Number and Severity of all Chronic Health Conditions (CHC) on the Risk of Negative Employment Transitions
eFigure. Flow Diagram for the Study Population Derivation

This supplemental material has been provided by the authors to give readers additional information about their work.

© 2024 Bhatt NS et al. *JAMA Network Open.*

eTable 1. Mapping of Employment Status Variables From Childhood Cancer Survivor Study (CCSS) and Behavioral Risk Factor Surveillance System (BRFSS) Cohorts

Categories of employment status created for this analysis	CCSS categories	BRFSS categories
Employed	Full-time work,<br>Part-time work	Employed for wages,<br>Self-employed
Health-related unemployment	Unable to work due to illness/ disability	Inability to work
Unemployed	Unemployed and looking for work	Out of work for more than 1 year; Out of work for less than 1 year
Not in labor force	Caring for home or family	Homemaker
	Student	Student
	Retired	Retired

© 2024 Bhatt NS et al. *JAMA Network Open.*

eTable 2. Sex Stratified Demographic and Treatment Characteristics of Childhood Cancer Survivors Diagnosed Between 1970 and 1986 and Aged 25 or more Years at the Time of Survey According to Their Response to Baseline and/or Follow-Up Surveys

Characteristic		Female survivors			
		Answered baseline and/or follow-up surveys			
		Answered either* (N=3980)		Answered both** (N=2243)	
		Median	Range	Median	Range
Age at diagnosis (years)		7	(0, 20)	9	(0, 20)
Age at first completed survey (years)		34	(25, 62)	33	(25, 53)
Years since diagnosis to first completed survey		26.9	(16.2, 46.0)	24.7	(16.2, 34.4)
**Characteristic**	**Category**	**N**	**%**	**N**	**%**
Age at diagnosis (years)	0-4	1514	38	615	27
	5-9	851	21	517	23
	10-14	865	22	589	26
	15-20	750	19	522	23
Race/ Ethnicity	White non-Hispanic	3552	89	1967	92
	Other	428	11	181	8
	Unknown	0		95	
Health insurance status	Yes	3598	91	1934	87
	No	354	9	180	8
	Canadian resident	5	0.1	114	5
	Not reported	23		15	
Primary cancer diagnosis	Acute lymphoblastic leukemia	1216	31	662	30
	Acute myeloid leukemia	122	3	59	3
	Astrocytoma	307	8	161	7
	Medulloblastoma	81	2	48	2
	Hodgkin lymphoma	565	14	358	16
	Non-Hodgkin lymphoma	198	5	126	6
	Kidney (Wilms)	407	10	197	9
	Neuroblastoma	281	7	110	5
	Sarcomas	704		461	
	Soft tissue sarcoma	357	9	224	10
	Ewing sarcoma	107	3	69	3
	Osteosarcoma	240	6	168	7
	Other diagnoses	99		61	
	Other leukemia	26	0.6	17	0.8
	Other CNS tumors	58	1	28	1
	Other bone tumors	15	0.4	16	0.7
Treatment combinations	No Surgery, Chemotherapy, or Radiation	8	0.2	3	0.1
	Surgery only	284	8	166	8
	Chemotherapy only	352	10	145	7
	Radiation only	9	0.2	4	0.2
	Surgery + Chemotherapy	606	17	333	16
	Surgery + Radiation	478	13	296	14
	Chemotherapy + Radiation	644	18	401	19
	Surgery + Chemotherapy + Radiation	1215	34	751	36
Employment Status from first questionnaire	Full-time work	2271	57	1337	60
	Part-time work	555	14	324	15
	Caring for home or family	450	11	272	12
	Unemployed and looking for work	162	4	81	4

© 2024 Bhatt NS et al. *JAMA Network Open.*

	Health-related unemployment	463	12	159	7
	Retired	6	0	3	0.1
	Student	63	2	34	2
	Unemployed, cause not specified	10	0	7	0.3
	Not reported	0		26	
Age at first survey (years)	25-34	2208	55	1260	56
	35-44	1443	36	846	38
	≥45	329	8	137	6

Characteristic		Male survivors			
		Answered baseline and/or follow-up surveys			
		Answered either* (N=3982)		Answered both** (N=2048)	
		Median	Range	Median	Range
Age at diagnosis (years)		7	(0, 20)	9	(0, 20)
Age at first completed survey (years)		34	(25, 62)	33	(25, 53)
Years since diagnosis to first completed survey		26.3	(16.1, 46.5)	24.4	(16.1, 34.0)
**Characteristic**	**Category**	**N**	**%**	**N**	**%**
Age at diagnosis (years)	0-4	1380	35	500	24
	5-9	953	24	534	26
	10-14	881	22	558	27
	15-20	768	19	456	22
Race/ Ethnicity	White non-Hispanic	3604	91	1799	93
	Other	378	9	144	7
	Unknown	0		105	
Health insurance status	Yes	3472	88	1733	85
	No	478	12	206	10
	Canadian resident	7	0.2	96	5
	Not reported	25		13	
Primary cancer diagnosis	Acute lymphoblastic leukemia	1153	29	572	28
	Acute myeloid leukemia	89	2	35	2
	Astrocytoma	267	7	137	7
	Medulloblastoma	100	3	46	2
	Hodgkin lymphoma	568	14	314	15
	Non-Hodgkin lymphoma	444	11	250	12
	Kidney (Wilms)	318	8	113	6
	Neuroblastoma	205	5	76	4
	Sarcomas	704		424	
	Soft tissue sarcoma	370	9	206	10
	Ewing sarcoma	114	3	72	4
	Osteosarcoma	220	6	146	7
	Other diagnoses	134		81	
	Other leukemia	37	0.9	23	1
	Other CNS tumors	84	2	51	2
	Other bone tumors	13	0.3	7	0.3
Treatment combinations	No Surgery, Chemotherapy, or Radiation	10	0.3	4	0.2
	Surgery only	248	7	141	8
	Chemotherapy only	172	5	70	4
	Radiation only	12	0.3	9	0.5
	Surgery + Chemotherapy	708	20	365	20
	Surgery + Radiation	401	11	245	13
	Chemotherapy + Radiation	317	9	184	10
	Surgery + Chemotherapy + Radiation	1622	46	839	45

© 2024 Bhatt NS et al. *JAMA Network Open.*

Employment status from first questionnaire	Full-time work	3179	80	1712	85
	Part-time work	206	5	91	5
	Caring for home or family	23	0.6	7	0.35
	Unemployed and looking for work	158	4	70	3
	Health-related unemployment	348	9	96	5
	Retired	6	0.2	4	0.20
	Student	52	1	36	2
	Unemployed, cause not specified	10	0.2	5	0.25
	Not reported	0		27	
Age at first survey (years)	25-34	2164	54	1160	57
	35-44	1483	37	780	38
	≥45	335	8	108	5

*Participants who responded to either baseline or follow-up surveys were restricted to those with available US address because of the comparison with BRFSS cohort
**Participants who responded to both baseline and follow-up surveys were not restricted to those with available US address

© 2024 Bhatt NS et al. *JAMA Network Open.*

eTable 3. Prevalence, Standardized Prevalence Ratio (SPR), and 95% CI of Employment Status Among Childhood Cancer Survivors Enrolled in CCSS Original Cohort Relative to BRFSS Rates According to Time of Follow-Up Survey

	**Full-time/ Part-time employment**				**Health-related unemployment**			
	**Females**		**Males**		**Females**		**Males**	
	**Prevalence**	**SPR (95% CI)**	**Prevalence**	**SPR (95% CI)**	**Prevalence**	**SPR (95% CI)**	**Prevalence**	**SPR (95% CI)**
Baseline (2002-2004)	2215/3076 (71.3)	1.01 (0.98, 1.03)	2753/3196 (85.3)	0.96 (0.94, 0.97)	328/3076 (11.6)	3.78 (3.37, 4.23)	244/3196 (8.1)	3.12 (2.71, 3.60)
Follow-up (2014-2016)	1933/2852 (64.8)	0.94 (0.90, 0.98)	2079/2557 (77.3)	0.92 (0.89, 0.95)	422/2852 (17.2)	2.23 (1.97, 2.51)	320/2557 (17.1)	2.61 (2.24, 3.03)
P-value	<0.001	<0.001	<0.001	0.020	<0.001	<0.001	<0.001	0.002
	**Unemployed and looking for work**				**Not part of the labor Force**			
	**Females**		**Males**		**Females**		**Males**	
	**Prevalence**	**SPR (95% CI)**	**Prevalence**	**SPR (95% CI)**	**Prevalence**	**SPR (95% CI)**	**Prevalence**	**SPR (95% CI)**
Baseline (2002-2004)	132/3076 (4.3)	0.81 (0.67, 0.97)	135/3196 (4.5)	0.87 (0.72, 1.10)	401/3076 (12.8)	0.61 (0.55, 0.67)	64/3196 (2.1)	0.68 (0.50, 0.92)
Follow-up (2014-2016)	115/2852 (5.2)	0.97 (0.66, 1.40)	97/2557 (3.7)	0.64 (0.48, 0.90)	382/2852 (12.8)	0.71 (0.62, 0.81)	61/2557 (2.0)	0.55 (0.41, 0.73)
P-value	0.85	0.55	0.28	0.12	0.66	0.003	0.98	0.38

*Percents and calculated SPRs are weighted to account for sampling and dropout. N's are not weighted

© 2024 Bhatt NS et al. *JAMA Network Open.*

eTable 4. Characteristics of Childhood Cancer Survivors Diagnosed Between 1970 and 1986 and Aged 25 or more years at the Time of Survey Who Reported Health-Related Unemployment at Baseline

Females reporting health-related unemployment at baseline

Characteristic	Categories	Either answered to follow-up or died before follow-up** (N=250)		Reporting health-related unemployment at follow-up (N=124)		Those who died before follow-up (N=97)	
		**Median**	**(range)**	**Median**	**(range)**	**Median**	**(range)**
Age at diagnosis (years)		9	(0-20)	7	(0-20)	10	(0-20)
Age at baseline survey (years)		34	(25-53)	33	(25-50)	37	(25-53)
Years since diagnosis to baseline		26.3	(16.5-34.1)	25.7	(16.5-34.1)	27.7	(17.4-34.1)
Age at follow-up (years)		-		45	(34-65)	-	
Years between baseline & follow-up		-		11.4	(9.4-13.5)	-	
**Characteristic**	**Categories**	**N**	**(%)**	**N**	**(%)**	**N**	**(%)**
Race/ Ethnicity	White not Hispanic	210	(89.4)	104	(90.4)	84	(91.3)
	Other	25	(10.6)	11	(9.6)	8	(8.7)
	Not reported	15		9		5	
Health insurance at baseline	Yes	210	(85.0)	104	(85.2)	82	(85.4)
	No	27	(10.9)	14	(11.5)	9	(9.4)
	Canadian resident	10	(4.7)	4	(3.3)	5	(5.2)
	Not reported	3		2		1	
Health insurance at follow-up	Yes	-		107	(88.4)	-	
	No	-		7	(5.8)	-	
	Canadian resident	-		7	(5.8)	-	
	Not reported	-		3		-	
Education	1-8 years (grade school)	11	(4.5)	8	(6.6)	3	(3.1)
	9-12 years (high school), not high school graduate	25	(10.1)	16	(13.1)	9	(9.4)
	Completed high school	72	(29.1)	37	(30.3)	25	(26.0)
	Post high school, not college	22	(8.9)	9	(7.4)	11	(11.5)
	Some college	62	(25.1)	31	(25.4)	24	(25.0)
	College graduate	45	(18.2)	17	(13.9)	18	(18.8)
	Post graduate level	10	(4.0)	4	(3.3)	6	(6.3)
	Not reported	3		2		1	
Marital status	Single (never married)	127	(51.0)	67	(54.5)	50	(51.5)
	Married	73	(29.3)	30	(24.4)	27	(27.8)
	Living as married	7	(2.8)	4	(3.3)	3	(3.1)
	Widowed	4	(1.6)	3	(2.4)	0	(0)
	Divorced	28	(11.2)	15	(12.2)	12	(12.4)
	Separated	10	(4.0)	4	(3.3)	5	(5.2)
	Not reported	1		1		0	

© 2024 Bhatt NS et al. *JAMA Network Open.*

Status at follow-up	Full-time work	12	(4.8)	-		-	
	Part-time work	4	(1.6)	-		-	
	Caring for home or family	8	(3.2)	-		-	
	Unemployed and looking for work	4	(1.6)	-		-	
	Health-related unemployment	124	(49.6)	124	(100)	-	
	Unemployed, cause not specified	1	(0.4)	-		-	
	Missing employment	0	(0)	-			
	died prior to follow-up	97	(38.8)	-		97	(100)
Primary cancer diagnosis	Acute lymphoblastic leukemia	64	(25.6)	45	(36.3)	18	(18.6)
	Acute myeloid leukemia	8	(3.2)				
	Astrocytoma	44	(17.6)	35	(28.2)	25	(25.8)
	Medulloblastoma	11	(4.4)				
	Hodgkin lymphoma	26	(10.4)	3	(2.4)	22	(22.7)
	Non-Hodgkin lymphoma	9	(3.6)	4	(3.2)	5	(5.2)
	Kidney (Wilms) tumors	8	(3.2)	3	(2.4)	4	(4.1)
	Neuroblastoma	9	(3.6)	5	(4)	3	(3.1)
	Sarcoma	62		13	(10.5)	10	(10.3)
	Soft tissue sarcoma	28	(11.2)				
	Ewings sarcoma	12	(4.8)				
	Osteosarcoma	22	(8.8)				
	Other cancers	9		16	(12.9)	10	(10.3)
	Other leukemias	1	(0.4)				
	Other CNS tumors	8	(3.2)				
	Other bone tumors	0	(0)				
Treatment combinations	No surgery, chemotherapy, or radiation	0	(0)	10	(8.6)	0	(0)
	Surgery only	21	(9.2)	5	(4.3)	6	(7.1)
	Chemotherapy only	9	(3.9)	0	(0)	2	(2.4)
	Radiation only	1	(0.4)	0	(0)	1	(1.2)
	Surgery + Chemotherapy	29	(12.7)	15	(12.9)	6	(7.1)
	Surgery + Radiation	44	(19.2)	25	(21.6)	18	(21.2)
	Chemotherapy + Radiation	44	(19.2)	28	(24.1)	10	(11.8)
	Surgery + Chemotherapy + Radiation	81	(35.4)	33	(28.5)	42	(49.4)
	Missing	21		8		12	
Severity and number of chronic health conditions, any type, before baseline	No grade 2-4	24	(9.6)	12	(9.7)	9	(9.3)
	One grade 2, no grade 3-4	17	(6.8)	9	(7.3)	6	(6.2)
	Two or more grade 2, no grade 3-4	17	(6.8)	6	(4.8)	6	(6.2)
	One grade 3-4	77	(30.8)	43	(34.7)	24	(24.7)
	Two or more grade 3-4	115	(46.0)	54	(43.6)	52	(53.6)
Severity and number of chronic health conditions between baseline and follow-up*	No grade 2-4			43	(34.7)	40	(41.2)
	One grade 2, no grade 3-4			29	(23.4)	0	(0)
	Two or more grade 2, no grade 3-4			11	(8.9)	1	(1.0)
	One grade 3-4			27	(21.8)	46	(47.4)
	Two or more grade 3-4			14	(11.3)	10	(10.3)

© 2024 Bhatt NS et al. *JAMA Network Open.*

Grade 3-4 CHCs occurred before baseline	Subsequent neoplasm	40	(16.0)	15	(12.1)	21	(21.6)
	Hearing	35	(14.0)	21	(16.9)	13	(13.4)
	Vision	34	(13.6)	21	(16.9)	11	(11.3)
	Endocrine	50	(20.0)	19	(15.3)	29	(29.9)
	Respiratory	14	(5.6)	6	(4.8)	7	(7.2)
	Cardiac	69	(27.6)	24	(19.4)	35	(36.1)
	GI	17	(6.8)	7	(5.6)	9	(9.3)
	Renal	6	(2.4)	2	(1.6)	3	(3.1)
	Musculoskeletal	33	(13.2)	18	(14.5)	7	(7.2)
	Neurologic	90	(24.5)	43	(34.7)	23	(23.7)
Grade 3-4* CHCs occurred between baseline and follow-up	Subsequent neoplasm			7	(5.6)	23	(23.7)
	Hearing			4	(3.2)	2	(2.1)
	Vision			1	(0.8)	1	(1.0)
	Endocrine			4	(3.2)	3	(3.1)
	Respiratory			4	(3.2)	7	(7.2)
	Cardiac			19	(15.3)	15	(15.5)
	GI			1	(0.8)	6	(6.2)
	Renal			2	(1.6)	0	(0)
	Musculoskeletal			4	(3.2)	0	(0)
	Neurologic			7	(5.6)	6	(6.2)

Males reporting health-related unemployment at baseline

Characteristic	Categories	Either answered to follow-up or died before follow-up** (N=215)		Reporting health-related unemployment at follow-up (N=75)		Those who died before follow-up (N=122)	
		Median	(Range)	Median	(Range)	Median	(Range)
Age at diagnosis (years)		10	(0-20)	10	(1-20)	10	(0-20)
Age at baseline survey (years)		35	(25-52)	35	(25-47)	35	(25-52)
Years since diagnosis to survey		25.9	(16.7-33.2)	24.8	(16.7-32.9)	26.9	(17.0-33.2)
Age at follow-up survey (years)		-		45	(34-65)	-	
Years between baseline and follow-up		-				-	
**Characteristic**	**Categories**	**N**	**(%)**	**N**	**(%)**	**N**	**(%)**
Race	White not Hispanic	169	(84.1)	61	(85.9)	94	(83.2)
	Other	32	(15.9)	10	(14.1)	19	(16.8)
	Not reported	14		4		9	
Health insurance at baseline	Yes	162	(76.8)	57	(76)	95	(80.5)
	No	33	(15.6)	11	(14.7)	15	(12.7)
	Canadian resident	16	(7.6)	7	(9.3)	8	(6.8)
	Not reported	3		0		4	
Health insurance at follow-up	Yes	-		62	(83.8)	-	
	No	-		3	(4.1)	-	
	Canadian resident	-		9	(12.2)	-	

© 2024 Bhatt NS et al. *JAMA Network Open.*

	Not reported	-		1		-	
Education	1-8 years (grade school)	11	(5.1)	2	(2.7)	9	(7.4)
	9-12 years (high school), not high school graduate	22	(10.3)	8	(10.8)	13	(10.7)
	Completed high school	73	(34.1)	28	(37.8)	39	(32.0)
	Post high school, not college	27	(12.6)	8	(10.8)	17	(13.9)
	Some college	47	(22.0)	17	(23)	23	(18.9)
	College graduate	28	(13.1)	9	(12.2)	17	(13.9)
	Post graduate level	6	(2.8)	2	(2.7)	4	(3.3)
	Not reported	1		1		0	
Marital status	Single (never married)	140	(65.1)	50	(66.7)	81	(66.4)
	Married	43	(20.0)	17	(22.7)	23	(18.9)
	Living as married	8	(3.7)	1	(1.3)	5	(4.1)
	Widowed	0	(0)	0	(0)	0	(0)
	Divorced	18	(8.4)	6	(8)	10	(8.2)
	Separated	6	(2.8)	1	(1.3)	3	(2.5)
	Not reported	0		0		0	
Status at follow-up	Full-time work	10	(4.7)	-		-	
	Part-time work	2	(0.9)	-		-	
	Caring for home or family	2	(0.9)	-		-	
	Unemployed and looking for work	2	(0.9)	-		-	
	Health-related unemployment	75	(34.9)	75	(100)	-	
	Retired	1	(0.5)	-		-	
	Unemployed, cause not specified	1	(0.5)	-		-	
	Missing employment data	0	(0)	-		-	
	died prior to follow-up	122	(56.7)	-		122	(100)
Primary cancer diagnosis	Acute lymphoblastic leukemia	33	(15.3)	21	(28)	14	(11.5)
	Acute myeloid leukemia	5	(2.3)				
	Astrocytoma	40	(18.6)	25	(33.3)	46	(37.7)
	Medulloblastoma	22	(10.2)				
	Hodgkin lymphoma	28	(13.0)	4	(5.3)	24	(19.7)
	Non-Hodgkin lymphoma	17	(7.9)	3	(4.0)	8	(6.6)
	Kidney (Wilms) tumors	8	(3.7)	1	(1.3)	6	(4.9)
	Neuroblastoma	2	(0.9)	1	(1.3)	0	(0)
	Sarcomas	48		9	(12.0)	12	(9.8)
	Soft tissue sarcoma	21	(9.8)				
	Ewings sarcoma	8	(3.7)				
	Osteosarcoma	19	(8.8)				
	Other cancers	12		11	(14.7)	12	(9.8)
	Other leukemias	1	(0.5)				
	Other CNS tumors	10	(4.7)				
	Other bone tumors	1	(0.5)				
Treatment combinations	No surgery, chemotherapy, or radiation	0	(0)	0	(0)	0	(0)

© 2024 Bhatt NS et al. *JAMA Network Open.*

	Surgery only	10	(5.3)	7	(9.9)	2	(2.0)
	Chemotherapy only	7	(3.7)	3	(4.2)	3	(3.0)
	Radiation only	1	(0.5)	0	(0)	1	(1.0)
	Surgery + Chemotherapy	21	(11.2)	10	(14.1)	7	(6.9)
	Surgery + Radiation	44	(23.4)	15	(21.1)	28	(27.7)
	Chemotherapy + Radiation	16	(8.5)	8	(11.3)	6	(5.9)
	Surgery + Chemotherapy + Radiation	89	(47.3)	28	(39.4)	54	(53.5)
	Missing	27		4		21	
Severity and number of chronic health conditions, any type, prior to baseline	No grade 2-4	25	(11.6)	8	(10.7)	12	(9.8)
	One grade 2, no grade 3-4	14	(6.5)	8	(10.7)	5	(4.1)
	Two or more grade 2, no grade 3-4	14	(6.5)	5	(6.7)	8	(6.6)
	One grade 3-4	70	(32.6)	28	(37.3)	38	(31.1)
	Two or more grade 3-4	92	(42.8)	26	(34.7)	59	(48.4)
Severity and number of chronic health conditions, any type, between baseline and follow-up*	No grade 2-4			23	(30.7)	50	(41.0)
	One grade 2, no grade 3-4			14	(18.7)	4	(3.3)
	Two or more grade 2, no grade 3-4			7	(9.3)	0	(0)
	One grade 3-4			18	(24)	54	(44.3)
	Two or more grade 3-4			13	(17.3)	14	(11.5)
Grade 3-4 CHCs occurred by baseline	Subsequent neoplasm	26	(12.1)	8	(10.7)	17	(13.9)
	Hearing	38	(17.7)	8	(10.7)	27	(22.1)
	Vision	26	(12.1)	9	(12)	15	(12.3)
	Endocrine	24	(11.2)	11	(14.7)	12	(9.8)
	Respiratory	9	(4.2)	2	(2.7)	7	(5.7)
	Cardiac	53	(24.7)	16	(21.3)	34	(27.9)
	GI	12	(5.6)	3	(4)	7	(5.7)
	Renal	6	(2.8)	2	(2.7)	4	(3.3)
	Musculoskeletal	25	(11.6)	11	(14.7)	11	(9.0)
	Neurologic	49	(22.8)	17	(22.7)	30	(24.6)
Grade 3-4* CHC occurred between baseline and follow-up	Subsequent neoplasm			5	(6.7)	20	(16.4)
	Hearing			10	(13.3)	1	(0.8)
	Vision			3	(4)	1	(0.8)
	Endocrine			3	(4)	3	(2.5)
	Respiratory			1	(1.3)	9	(7.4)
	Cardiac			9	(12)	18	(14.8)
	GI			3	(4)	6	(4.9)
	Renal			3	(4)	7	(5.7)
	Musculoskeletal			3	(4)	0	(0)
	Neurologic			3	(4)	4	(3.3)

*Grade 3-5 conditions reported for survivors who died between baseline and follow-up; CHC= chronic health conditions
**Not including those alive at follow-up (assumed as of 11-30-2016) but did not return a questionnaire N=111 Females, N=82 Males

© 2024 Bhatt NS et al. *JAMA Network Open.*

eTable 5. Sex-Stratified Details on Overall and Organ-Specific Maximum Severity, Number, and Timing of Chronic Health Conditions of Childhood Cancer Survivors Who Reported Working Full-Time at Baseline and Responded to the Follow-Up Survey

Characteristic		Categories	Females		Males	
			N	%	N	%
Vision	Before baseline	No grade 2-4	1233	92	1586	93
		Maximum grade 2	61	5	66	4
		Maximum grade 3-4	43	3	60	4
	Between baseline and follow-up	No grade 2-4	1296	97	1647	96
		Maximum grade 2	18	1	32	2
		Maximum grade 3-4	23	2	33	2
Endocrine	Before baseline	No grade 2-4	886	66	1335	78
		Maximum grade 2	219	16	235	14
		Maximum grade 3-4	232	17	142	8
	Between baseline and follow-up	No grade 2-4	1009	75	1439	84
		Maximum grade 2	228	17	176	10
		Maximum grade 3-4	100	7	97	6
Respiratory	Before baseline	No grade 2-4	1222	91	1634	95
		Maximum grade 2	94	7	61	4
		Maximum grade 3-4	21	2	17	1.0
	Between baseline and follow-up	No grade 2-4	1305	98	1691	99
		Maximum grade 2	24	2	11	0.6
		Maximum grade 3-4	8	1	10	0.6
Cardiac	Before baseline	No grade 2-4	1008	75	1295	76
		Maximum grade 2	213	16	279	16
		Maximum grade 3-4	116	9	138	8
	Between baseline and follow-up	No grade 2-4	944	71	1149	67
		Maximum grade 2	265	20	380	22
		Maximum grade 3-4	128	10	183	11
Musculoskel etal	Before baseline	No grade 2-4	1231	92	1569	92
		Maximum grade 2	2	0.2	4	0.2
		Maximum grade 3-4	104	8	139	8
	Between baseline and follow-up	No grade 2-4	1319	99	1687	99
		Maximum grade 2	0	0	0	0
		Maximum grade 3-4	18	1	25	1
N eu rol og	Before baseline	No grade 2-4	1183	88	1547	90
		Maximum grade 2	107	8	111	6

© 2024 Bhatt NS et al. *JAMA Network Open.*

		Maximum grade 3-4	47	4	54	3
	Between baseline and follow-up	No grade 2-4	1277	96	1627	95
		Maximum grade 2	41	3	65	4
		Maximum grade 3-4	19	1	20	1
Severity and number of chronic health conditions, any type, before baseline		No grade 2-4	215	16	303	18
		One grade 2, no grade 3-4	140	10	150	9
		Two or more grade 2, no grade 3-4	351	26	446	26
		One grade 3-4	191	14	147	9
		Two or more grade 3-4	440	33	666	39
Severity and number of chronic health conditions between baseline and follow-up		No grade 2-4	268	20	326	19
		One grade 2, no grade 3-4	126	9	159	9
		Two or more grade 2, no grade 3-4	275	21	282	16
		One grade 3-4	127	10	158	9
		Two or more grade 3-4	541	40	787	46

© 2024 Bhatt NS et al. *JAMA Network Open.*

eTable 6. Overall Impact of Number and Severity of all Chronic Health Conditions (CHC) on the Risk of Negative Employment Transitions

Risk-factor	Categories	Females		Males	
		PR (95% CI)	P-value	PR (95% CI)	P-value
**Age at diagnosis**	0-4	1.02 (0.71, 1.47)	0.92	1.42 (0.94, 2.14)	0.10
	5-9	1.01 (0.72, 1.43)	0.96	1.67 (1.16, 2.40)	0.006
	10-14	0.89 (0.66, 1.21)	0.45	1.50 (1.08, 2.08)	0.016
	≥15	1	-	1	-
**Race**	Others	1.17 (0.88, 1.55)	0.29	0.95 (0.67, 1.36)	0.80
	White not Hispanic	1.0	-	1.0	-
**Age at follow-up**	<45	1.14 (0.88, 1.49)	0.31	0.81 (0.61, 1.07)	0.13
	≥45	1.0	-	1.0	-
**CHC occurred before baseline**	No grade 2-4	1.0	-	1.0	-
	One grade 2, no grade 3-4	0.80 (0.56, 1.16)	0.25	1.10 (0.75, 1.63)	0.62
	Two or more grade 2, no grade 3-4	1.55 (1.12, 2.13)	0.008	1.53 (1.02, 2.30)	0.04
	One grade 3-4	1.00 (0.75, 1.34)	0.99	1.40 (1.02, 1.92)	0.037
	Two or more grade 3-4	1.41 (1.04, 1.90)	0.026	2.99 (2.16, 4.14)	<0.001
**CHC occurred between baseline and follow-up**	No grade 2-4	1.0	-	1.0	-
	One grade 2, no grade 3-4	1.03 (0.75, 1.42)	0.84	0.67 (0.43-1.05)	0.08
	Two or more grade 2, no grade 3-4	1.11 (0.73, 1.69)	0.62	1.89 (1.30-2.74)	<0.001
	One grade 3-4	1.40 (1.07, 1.84)	0.015	1.61 (1.16-2.22)	0.004
	Two or more grade 3-4	2.32 (1.73, 3.11)	<0.001	3.19 (2.37-4.28)	<0.001

PR= Prevalence Ratio, CI= confidence interval

© 2024 Bhatt NS et al. *JAMA Network Open.*

eFigure. Flow Diagram for the Study Population Derivation

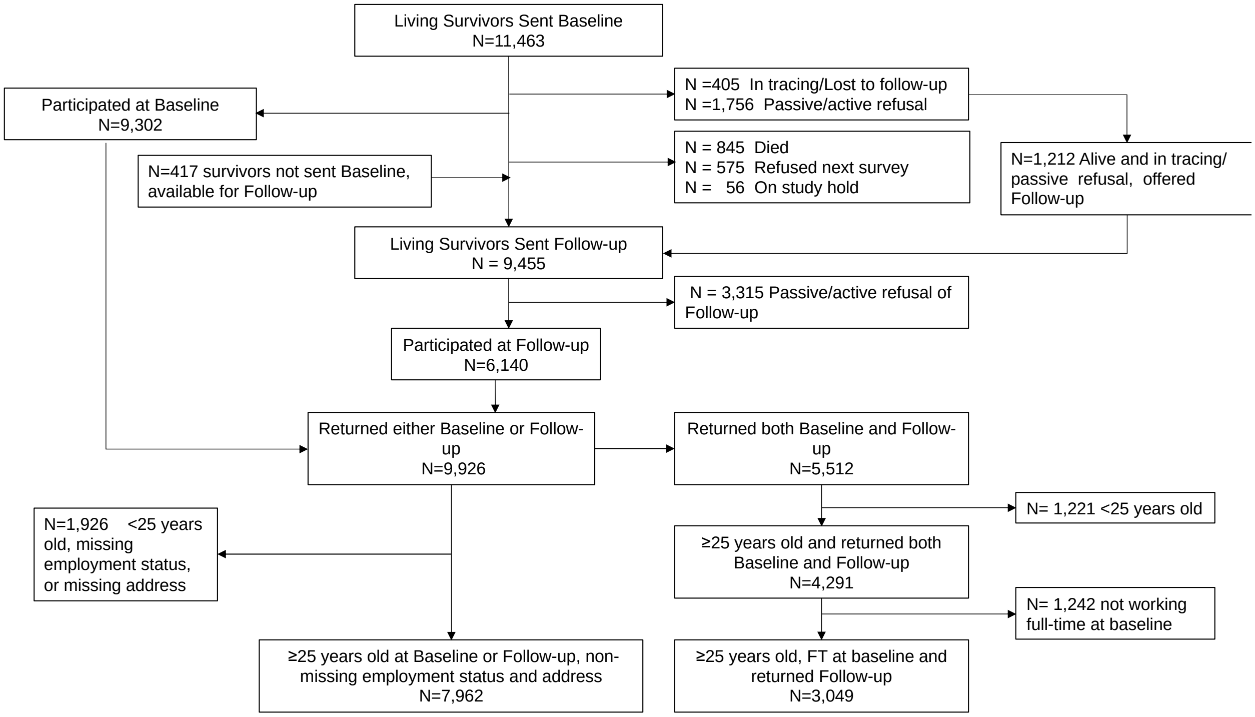

© 2024 Bhatt NS et al. *JAMA Network Open.*
